# Integrated Triboelectric Nanogenerators in the Era of the Internet of Things

**DOI:** 10.1002/advs.201802230

**Published:** 2019-09-30

**Authors:** Abdelsalam Ahmed, Islam Hassan, Maher F. El‐Kady, Ali Radhi, Chang Kyu Jeong, Ponnambalam Ravi Selvaganapathy, Jean Zu, Shenqiang Ren, Qing Wang, Richard B. Kaner

**Affiliations:** ^1^ School of Mechanical and Industrial Engineering University of Toronto Toronto ON M5S 3G8 Canada; ^2^ Department of Mechanical Engineering McMaster University Hamilton ON L8S 4L8 Canada; ^3^ School of Biomedical Engineering McMaster University Hamilton ON L8S 4L8 Canada; ^4^ Department of Chemistry and Biochemistry and California NanoSystems Institute University of California Los Angeles (UCLA) Los Angeles CA 90095 USA; ^5^ Department of Materials Science and Engineering UCLA Los Angeles CA 90095 USA; ^6^ Division of Advanced Materials Engineering Chonbuk National University Jeonju Jeonbuk 54896 Republic of Korea; ^7^ Schaefer School of Engineering and Science at Stevens Institute of Technology Hoboken NJ 07030 USA; ^8^ Department of Mechanical and Aerospace Engineering and Research and Education in Energy Environment and Water (RENEW) Institute University at Buffalo The State University of New York Buffalo NY 14260 USA; ^9^ Department of Materials Science and Engineering The Pennsylvania State University University Park PA 16802 USA

**Keywords:** blue energy, energy storage, Internet of Things (IoT), power management, smart cities, triboelectric nanogenerators

## Abstract

Since their debut in 2012, triboelectric nanogenerators (TENGs) have attained high performance in terms of both energy density and instantaneous conversion, reaching up to 500 W m^−2^ and 85%, respectively, synchronous with multiple energy sources and hybridized designs. Here, a comprehensive review of the design guidelines of TENGs, their performance, and their designs in the context of Internet of Things (IoT) applications is presented. The development stages of TENGs in large‐scale self‐powered systems and technological applications enabled by harvesting energy from water waves or wind energy sources are also reviewed. This self‐powered capability is essential considering that IoT applications should be capable of operation anywhere and anytime, supported by a network of energy harvesting systems in arbitrary environments. In addition, this review paper investigates the development of self‐charging power units (SCPUs), which can be realized by pairing TENGs with energy storage devices, such as batteries and capacitors. Consequently, different designs of power management circuits, supercapacitors, and batteries that can be integrated with TENG devices are also reviewed. Finally, the significant factors that need to be addressed when designing and optimizing TENG‐based systems for energy harvesting and self‐powered sensing applications are discussed.

## Graphical abstract

**Self‐powered and integrated triboelectric nanogenerators** are comprehensively reviewed based on blue energy, their performance, and hybrid designs under the context of large‐scale systems and Internet of Things applications.

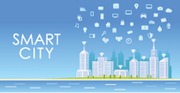

## 1 Introduction

Undeniable environmental changes in the form of climate change and air pollution are happening due to an exponential surge in the consumption of fossil fuels.^1^ To address this ever‐growing disaster, many approaches for generating power from renewable and sustainable sources, such as wind, wave, and solar energy, have been considered.^2^, ^3^, ^4^ These efforts focus on the use of massive farms of power generating devices that can harvest this renewable energy to work across modern electric grid systems. Besides, the variable and intermittent nature of solar and wind energy is inevitable and entails charge storage as a part of the energy harvesting system.

In recent years, the triboelectric nanogenerator (TENG) has developed as a capable concept for the use of multiple forms of renewable energy in the ambient environment.^4^, ^5^, ^6^, ^7^ A triboelectric nanogenerator relies on contact electrification coupled with electrostatic induction between two media for energy conversion from dynamic stimuli. First introduced by the Wang research group in 2012, the electrical output of a TENG is generated from the discharge of amassed static charges.^8^ The amassed static charges are the result of an imbalance between negative and positive charges in an object. These charges can build up on the surface of an object until they can be released or discharged. Compared to other energy harvesting techniques such as piezoelectric devices that can be used in a similar fashion, TENGs have been suggested to have higher energy harvesting efficiencies in the low‐frequency range.^9^ This phenomenon is particularly vital because, for ambient environmental sources, most of the surrounding media oscillates with low to medium frequency motion.

Many efforts have been devoted to exploring advanced TENG designs by inducing electron flow from coalescing electrostatic induction during contact electrification. These designs have attracted interest for microscale energy harvesting devices.^10^, ^11^ The employment of TENGs for self‐powered sensing applications seems to have had a high impact in promoting smart cities and IoT network. As such, TENGs have received significant attention because they can provide multifunctional power generation from multiple sustainable sources, with blue energy at the forefront of current TENG research.^6^, ^12^, ^13^ It is worth noting that the hydrodynamic energy from waves, tidal as well as wind currents has played a significant role in blue energy harvesting. Consequently, several TENG designs have been optimized to be quite efficient, showing great opportunities for multimode energy harvesting from this blue energy medium.^2^, ^14^, ^32^ The adaptive and integrative features of TENGs can provide significant assistance to wind‐based energy solutions with multiple hybrid blue energy sources, such as wind–wave, wind–solar, and water–solar. Therefore, the capability to harvest blue energy, especially with large networks under relatively weak water waves and air currents, puts TENGs at the forefront of developing the next generation of sustainable energy sources.

Thus far, the accumulated literature has highlighted the current progress of TENGs in self‐powered systems.^11^, ^15^ In contrast, in this review, we discuss the recorded current progress of TENGs as sustainable and large‐scale self‐charging power units (SCPUs). Thus, we briefly discuss the fundamental mechanisms related to TENGs. Then, we discuss the developed assessment models for characterizing, optimizing the performance of, and assessing the environmental impact of TENG devices. After that, the working mechanism and designs of water wave–based TENGs are discussed, followed by comparisons in terms of output power and other unique characteristics of the presented designs. Next, we discuss some of the mechanisms of wind‐based TENG harvesters and highlight some of their unique advantages and limitations. The subsequent section introduces some of the latest innovations in the field of hybrid energy harvesting and multimodal functionalities of blue energy TENGs. Then, we highlight the significant achievements in the fields of large‐scale energy harvesting and IoT devices operated by TENG‐based technologies. After reviewing the energy harvesting capabilities of the current state‐of‐the‐art TENG devices for electronic devices, we summarize the recent efforts made in designing both energy storage and power management systems that enable optimal SCPU performance in the IoT and smart city framework under sizable blue energy conversion from mechanical and/or electrical perspectives. According to these recent advances, some technological challenges such as low output power density, low energy conversion efficiency, packing issues, and how to overcome them will be addressed in a future outlook.

## 2 Principal Modes of Triboelectric Nanogenerators

A TENG is an energy scavenging device that converts exterior mechanical energy into electricity through a combination of the triboelectrification effect and electrostatic induction. Generally, a TENG is constructed by assembling two polymer layers, with thin metal films deposited on the top and bottom of the assembled device. Here, we introduce a short discussion on the different modes through which triboelectrification occurs. These modes include contact‐separation (CS), lateral‐sliding (LS), freestanding (FS), and single‐electrode (SE) modes.

### 2.1 Contact‐Separation Mode

**Figure 1:**
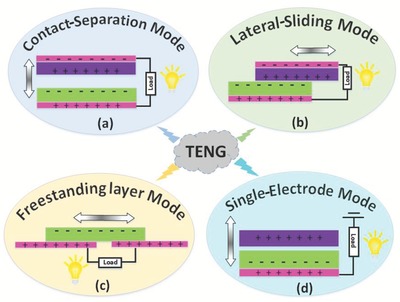
Fundamental modes of triboelectric nanogenerators: a) Contact‐separation mode. b) Lateral‐sliding mode. c) Freestanding mode. d) Single‐electrode mode.

In this approach, two terminals with opposite electrical polarities (positive and negative) are located on the top and bottom surfaces of the assembled structure, as shown in **Figure** **1**a. The two terminals have a small air gap between them. Exterior mechanical forces, such as water waves, cause these two electrodes to then come into contact, as demonstrated using a spring‐assisted TENG structure.^16^ As a result, a positive triboelectric charge is produced on one side, and a negative charge is generated on the other surface. In the release motion, these two surfaces are disconnected due to the removal of the external force, and the triboelectric charges electrostatically produce opposite charges on the terminals; consequently, a current is generated in the external circuit. Finally, the surfaces come into contact again, and the above cycle is repeated.

### 2.2 Lateral‐Sliding Mode

The triboelectric performance in this mode depends on the relative sidelong movement between the contact surfaces, with high potential for various mechanical applications.^17^ The power generation process in such TENG systems is represented in Figure 1b. In this approach, rather than immediate contact, two different terminals are rubbed in a tangential direction. When the two surfaces are engaged with or disengaged from one side, a periodical change in the contact area among the two sliding surfaces causes a lateral separation of the charge centers. Such electrical potential can then produce a flow of charges from one electrode to the other.

### 2.3 Freestanding Mode

The freestanding working mode, portrayed in Figure 1c, depends on a moving electrostatic induction for power generation. This mode is arguably considered the best design for TENGs, producing high efficiencies.^18^ In this design, a freestanding triboelectric layer is forced to slide along and/or contact a similar plane with other stationary terminals.^19^ Figure 1c demonstrates a TENG structure that relies on the relative movement between the electrode and dielectric material, in which triboelectrification and electrostatic induction enable development of an alternating electron flow driven between terminals.

### 2.4 Single‐Electrode Mode

Figure 1d shows the outline of a single electrode TENG, which relies on periodic changes in the contact surface area. The resulting electrostatic induction produces free electrons and creates a current across the external load.^20^ Such a mode can be used in various applications, including the direct development/electrical output of a triboelectric layer.

## 3 TENG Assessment Models

In addition to various TENG designs, guidelines for designing and optimizing TENGs have been developed through detailed finite element model “FEM” analysis, techno‐economic analysis, and environmental assessment.^22^, ^23^

### 3.1 Performance Assessment Model

**Figure 2:**
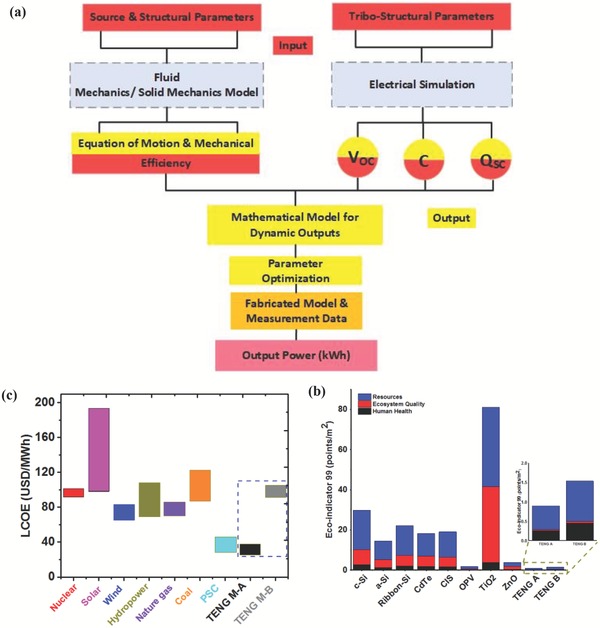
a) Flowchart of a semianalytical simulation model for a TENG energy harvester highlighting the effect of mechanical and electrical parameters on the optimized parameters. The input and output of the model are labeled in light blue and light red, respectively. b) Eco‐indicator results for 1 m^2^ of modules A and B. Reproduced with permission.^23^ Copyright 2017, The Royal Society of Chemistry. c) Comparison of LCOE for coal, natural gas, nuclear, wind, commercialized solar PV, hydropower, PSC, and TENG modules. Reproduced with permission.^23^ Copyright 2017, The Royal Society of Chemistry.

The developed models have been validated with experimental data by testing out different customized TENG designs.^22^, ^23^ In this process, the devices are thoroughly tested to find out appropriate TENG geometries that result in maximum power output, offer optimal efficiency, are cost‐effective, and are environmentally friendly (**Figure** **2**a).^23^ Important findings “such as structure and material optimization” were made, which can provide useful guidelines for future enhanced designs and simulations.^23^ Figure 2a shows the structure of a theoretical study carried out on TENGs. To examine a TENG as an energy harvester, an electrostatic FEM was built using COMSOL Multiphysics software. A three‐way coupling model, the “Tribo‐Fluid‐Solid Interaction Model,” was established, as outlined in Figure 2a, and the objective was to obtain the intersection between all three fields, that is, the crossing point between the three models: triboelectricity, solid mechanics, and fluid dynamics.^22^, ^23^

### 3.2 Life Cycle Assessment (LCA) Model

LCA studies focus on the global environmental impacts of devices at the consumer and product levels. Hence, evaluating the TENG's environmental profile and cost by performing environmental LCA and techno‐economic analysis is crucial, which will indicate whether TENGs are a promising candidate from these points of view. As shown in Figure 2a, point‐by‐point techno‐economic analyses and LCAs have been carried out for different TENG modules.^23^ For such techno‐economic and lifecycle assessments of two hypothetical cases of TENG modules, one module with elite effectiveness (TENG A) and another with lower productivity (TENG B) were manufactured with low‐cost materials. Furthermore, a comparison with solar, wind, nuclear, and hydrostatic technologies was performed, as shown in Figure 2b,c.^23^ This comparison considered both the levelized cost of electricity and the eco‐indicator of these technologies compared to TENGs. TENGs were concluded to exhibit a competitive trend with the abovementioned technologies.^23^ Moreover, the outcomes in comparison with other energy harvesting technologies, especially photovoltaics, have been considered, as shown in Figure 2c. As can be seen, TENG A has a superior ecological profile, bringing down the cost of fabrication, CO_2_ discharge, and energy payback period (EPBP) compared to TENG B.^23^ Based on these findings, we suggest that future research on TENGs concentrate on enhancing framework execution, improving their materials, and, more significantly, studying their life expectancy to understand their maximum capacity.^23^ This research effort focuses on identifying the complete design guidelines for TENGs for energy harvesting applications so that they can be utilized to develop enhanced output, sustainable and cost‐effective TENGs.

## 4 Hydrokinetic Energy Harvesting Schemes

**Figure 3:**
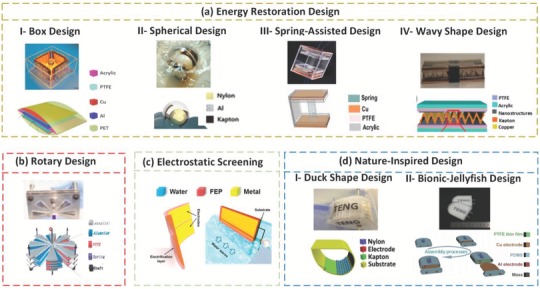
Hydrokinetic energy conversion schemes. a) Energy restoration mechanism. Real photos and schematic diagrams of fabricated TENGs: I) Box design; Reproduced with permission.^24^ Copyright 2015, American Chemical Society. II) Sphere design; Reproduced with permission.^25^ Copyright 2015, John Wiley & Sons. III) Spring‐assisted structure; Reproduced with permission.^16^ Copyright 2017, Elsevier. IV) Wavy shape; Reproduced with permission.^26^ Copyright 2014, Elsevier. b) Rotary design‐based TENG. A real photo and a schematic diagram of a multilayered disk. Reproduced with permission.^27^ Copyright 2014, Elsevier. c) Electrostatic asymmetric screening. The structural design of the liquid–solid electrification enabled generator; a schematic of the bent electrification layer with two electrodes on one side. Reproduced with permission.^28^ Copyright 2014, American Chemical Society. d) Nature‐Inspired TENG. Real photos and schematic diagrams of I) a duck shape design; Reproduced with permission.^5^ Copyright 2017, John Wiley and Sons; and II) a bionic‐jellyfish (bj) TENG; Reproduced with permission.^29^ Copyright 2017, Elsevier.

This section discusses the fundamental conversion mechanisms for incidental wave energy harvesting through exceptional structural and material design schemes. As shown in **Figure** **3**, several working mechanisms and designs of water wave–based TENGs are highlighted. We divide these different designs into four main approaches. In the first approach, the devices utilize an elastic element, such as a spring, to conserve energy and then convert this energy to kinetic energy. In the second one, wave energy is harvested by converting the water/wave motion to rotational motion between contact layers, thus generating energy due to triboelectrification phenomena. In the third approach, energy is scavenged from the water motion by using a plate, in which contact and separation occur between the water surface and the solid thin film. Finally, the last approach conserves water energy by using designs with nature‐like shapes to enhance device performance and stability. Comparisons of the presented designs in terms of the output power and other unique characteristics are summarized in **Table** **1**.

**Table 1:**
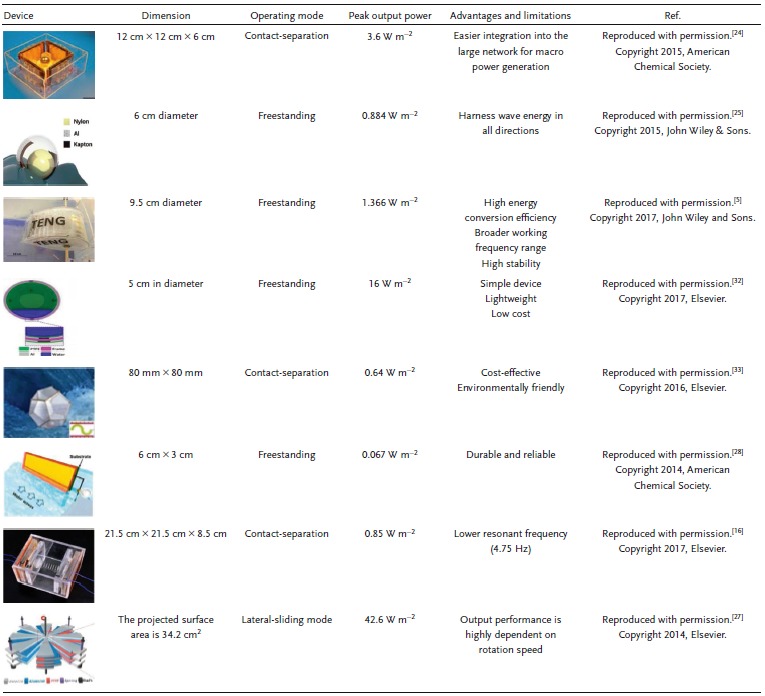
TENG‐based design configurations for wave energy harvesting

### 4.1 Energy Restoration–Based Mechanism

Energy restoration mechanisms refer to devices that can restore energy in potential form and release it in the next half cycle. For example, a 3D TENG structure with multiple interspersed layers of dielectric materials and the electrodes at the center, as proposed in the box ball design, is shown in Figure 3a(I). Both the upper and lower plates are made of polyethylene terephthalate, generally twisted by a heat treatment that activates powerful charges during contact/separation, thus utilizing the versatility of the film.^24^ A photo of an as‐created unit is provided in Figure 3a(I). The open‐circuit voltage and short‐circuit current generated at an acceleration of 10 m s^−2^ are 569.9 V, and 0.93 mA, respectively. Additionally, the peak power density at a load resistance of 1 MΩ is 0.26 mW cm^−2^. Moreover, a lightweight, rolling‐freestanding mode TENG with triboelectric layers (RF‐TENG) has been developed for harvesting energy from low‐recurrence tidal water waves. The TENG was first created by utilizing a moving nylon ball to contact a Kapton film encased in a 3D circular shell (Figure 3a(II)). The freestanding operation has an approximately linear relation with the charge exchange that guarantees appropriate electrical effectiveness, even under low‐magnitude vibrations.^25^ Furthermore, the moving shell significantly decreases the loss of energy through rubbing and enhances the energy conversion efficiency. This device can convey a large electron flow of up to 1 µA and a peak control power of up to 10 mW externally under real water wave conditions.^25^ Additionally, a spring‐connection type of TENG, in which two Cu/PTFE‐secured acrylic pieces connected by a spring are set between two Cu terminals and attached to two interior dividers of a crate (shown in Figure 3a(III)), was devised for reaping unidirectional wave energy.^16^ A photo of the TENG device as first produced is given in Figure 3a(III). By utilizing the spring, the charge collected by the TENG can be increased by 113.0% compared to the same system without the spring, and the induced electric yield or productivity can be enhanced by 150.3% compared to the same design without the spring. Moreover, the generated power volume density is 0.73 W m^−3,^ and the peak current is 70 µA, with a peak‐to‐peak voltage of 600 V. Alternatively, a TENG with wavy Cu‐Kapton‐Cu panels sandwiched between two‐level nanostructured PTFE films has been reported to utilize self‐restoring features without resorting to springs to operate in the LS mode.^26^ This design is considerably more efficient in the rubbing/sliding mode for power generation due to the large contact area, as depicted in Figure 3a(IV). The lightweight and high adaptability of the device guarantee that the generator can harvest small load amplitudes from the surrounding media except for under significant loads. Notably, the cost for such a device is calculated to be ≈$1.^26^ Moreover, this device can generate an open‐circuit voltage of 72 V, a short‐circuit current of 32 µA, and a high‐power density of 0.4 W m^−2^.

### 4.2 Rotary‐Based Mechanism

Several reports show that the optimum energy harvesting can be achieved from rotary motion through the design of a multilayer plate–like TENG.^27^ The device consists of segmentally organized circular sheets of triboelectric materials interspaced between electrode layers. A D‐shaped shaft coaxially converts the rotary motion for every rotor (turning the parts in each triboelectric layer pair), with synchronized phases for the segments, as portrayed in Figure 3b. Regarding the quantitative performance of this device, through a parallel network of four coordinated units in one multilayered plate TENG, the device can generate an improved short‐circuit current density (*J*_sc_) of 90.6 mA m^−2^ with a maximum energy density of 42.6 W m^−2^ (2.68 kW m^−3^) under a turning rate of 1000 rpm.^27^ The durability of such a rotating TENG design has also been studied.^30^ After continuously producing more than 10 million cycles of AC, the output current does not exhibit any measurable decay or degradation, which firmly proves the reliability and feasibility of this TENG in practical applications.^30^

### 4.3 Electrostatic Asymmetric Screening Mechanism

Liquid–Solid electrification enabled generator (LSEG) for collecting energy from an assortment of water movements has been presented.^28^ Having a planar structure (6 cm by 3 cm by 50 µm), one layer of the LSEG generates an ideal instantaneous output power of 0.12 mW, a current of 3 µA, and an open‐circuit voltage of 180 V at a speed of 0.5 m s^−1^. Nanowire‐based enhancements from polymeric substances play a crucial role in realizing such a high power yield.^28^ A fundamental unit of the LSEG is shown in Figure 3c. Different beneficial attributes, such as heat protection, radiation stability, and chemical dormancy, make fluorinated ethylene propylene an attractive and robust material for the networks during submersion operation. Additionally, patterned nanowires on the surface of the film give hydrophobic features, with the goal being that water is quickly repelled after sliding.

A drop of water has two types of energies that can be achieved in such a system: mechanical energy emerging from the impact of the falling drop and electrostatic energy induced due to contact electrification. In this capacity, a water TENG with a superhydrophobic miniaturized scale/nanostructured polytetrafluoroethylene (PTFE) surface was formulated to collect hydrokinetic energy from streaming water and water droplets.^31^ The yield of the water TENG produced from a 30 µL water drop can reach a maximum voltage of 9.3 V and a maximum current of 17 µA. A top energy yield of 145 µW is attained when the water TENG is associated with an external load resistor of 5 MΩ. The water TENG can also be used to harvest energy from streaming faucet water, and the current and instantaneous power densities achieved are 1.5 µA cm^−2^ and 20 mW cm^−2^, respectively.^31^

### 4.4 Nature‐Inspired Stimulated Mechanisms

Using an oscillating Salter's duck, a new TENG design was established so as to extract energy from random mechanical movement,^5^ as shown in Figure 3d(I). The device was shown to successfully harvest low‐frequency water waves and to withstand harsh environmental conditions thanks to its unique design that combines freestanding rolling mode with a duck‐shaped configuration. Owing to its restrictive shape, the device can pivot around a hub perpendicular to the wave direction. Correspondingly, the duck can demonstrate high productivities under ordinary operation and strong survivability under severe conditions.^34^ Additionally, the location of its center of gravity is such that the duck will return to its working position after the wave passes. The duck shape TENG device exhibits a novel reasonable upkeep for power generation needed for different wireless sensor network (WSN) applications, for example, condition checking of amphibious situations, atmospheric observations, and natural fire detection. A manufactured model of a duck shape freestanding triboelectric energy collector is shown in Figure 3d(I), and it can generate an electrical power up to 47.9 W m^−3^, a peak current of 65.5 µA, and an open‐circuit voltage of 300 V.^5^

Figure 3d(II) shows a simplistic design of a bionic‐jellyfish TENG (bjTENG) with polymeric thin films as the triboelectric material, which is adaptive and has a hermetic bundle and good resilience and elasticity, similar to a jellyfish. The versatility of the charge partition within this flexible bionic structure is based on the fluid pressure‐induced contact separation of the triboelectric layers. This device can yield 143 V and 11.8 mA m^−2^ under a low frequency up to 0.75 Hz at a water depth of 60 cm. This is enough power supply for a large number of green LEDs and/or a temperature sensor.^29^

## 5 Wind Energy Harvesting Schemes

**Figure 4:**
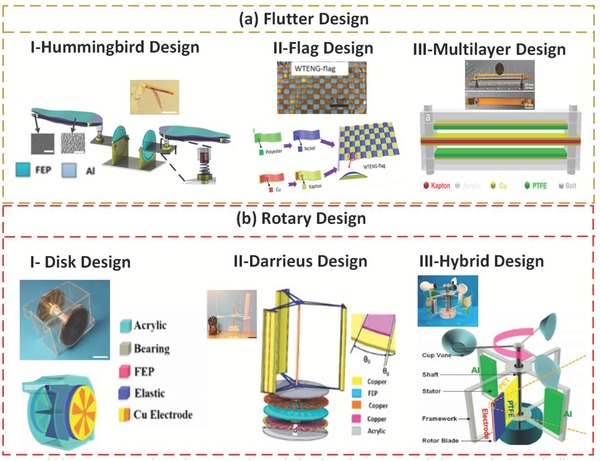
Wind energy conversion schemes. a) Flutter design‐based TENGs. Real photos and schematic diagrams of fabricated TENGs: I) Hummingbird TENG (H‐TENG); Reproduced with permission.^4^ Copyright 2017, Nature Publishing Group; II) Flag TENG. Reproduced with permission.^39^ Copyright 2016, American Chemical Society; III) Multilayer flutter TENG. Reproduced with permission.^41^ Copyright 2015, American Chemical Society. b) Rotary design‐based TENGs. Real photos and schematic diagrams of fabricated TENGs: I) Rotary disk TENG; Reproduced with permission.^42^ Copyright 2015, Elsevier; II) Darrieus rotary TENG; Reproduced with permission.^19^ Copyright 2017, Elsevier; III) Hybrid TENG. Reproduced with permission.^43^ Copyright 2013, American Chemical Society.

Wind is another vigorous source for clean energy that can be efficiently harvested using TENGs. TENGs have been widely used for harvesting energy from small amplitude wind streams, exploiting all of the translational, rotational, and oscillating motions of abundant weak wind currents.^35^ Here, as shown in **Figure** **4**, we discuss some of the more notable designs and mechanisms of wind‐based TENG harvesters. One of the distinctive approaches used in the literature to scavenge wind energy is the flutter mechanism, which has been used many times in different designs and structures. The other approach we highlight here is the traditional design based on rotational motion, such as the conventional wind turbine. **Table** **2** highlights some of the unique advantages and limitations of different designs for wind energy harvesting based on TENGs.

**Table 2:**
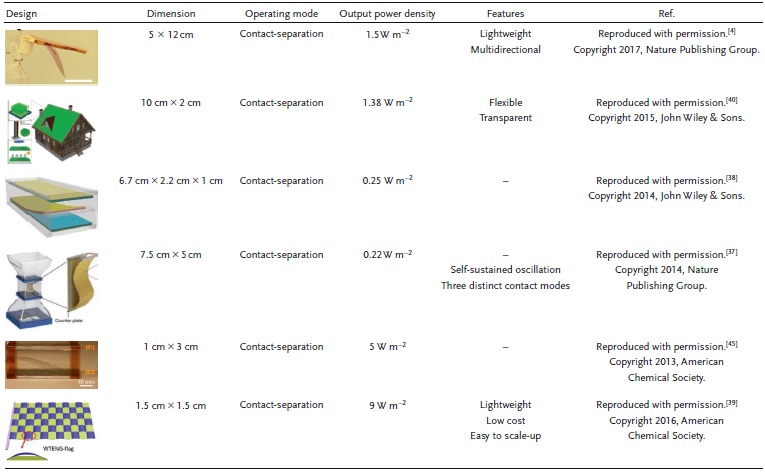
TENG‐based design configurations for wind energy harvesting

### 5.1 Flutter‐Based Mechanism

Figure 4a(I) shows that bioinspired innovations have excellent potential for advanced energy harvesting from maintainable and renewable resources.^4^ Driven by a hummingbird‐wing structure, a robust, lightweight TENG for wind energy harvesting using the flutter mechanism was introduced. This mechanism is restricted between two surfaces, generating power from contact electrification during fluttering. The hummingbird TENG (H‐TENG) device weighs 10 g, making it one of the lightest reported TENG devices. With a six TENG system, the connected apparatus realizes a 1.5 W m^−2^ maximum electrical density at a 7.5 m s^−1^ wind speed, with an observed linear increment in the charge rate with more TENG units. In addition, the open‐circuit voltage (*V*_OC_) and short‐circuit current (*I*_SC_) generated from one unit are 300 V and 100 µA, respectively. The capacity of the H‐TENG systems to power up IoT devices using wind resources under ambient conditions has been demonstrated. Additionally, the rippling response of a flexible wing‐like structure or flags of various textures and shapes^36^ attached to rigid plates can be used for effective vibration energy harvesting using a triboelectric generator. Moreover, the design of a flutter‐driven TENG depends on the metal separator structure, and the PTFE utilized as an encasement, which tends to pick up electrons on its surface by sliding along the surface.^37^ This 7.5 cm × 5 cm FTENG produced momentary voltage, current, and energy yields of 250 V, 70 µA, and 17.5 mW, respectively, at the approaching stream speed of 22 m s^−1^. The FTENG with a double plate design can completely charge a 100 µF capacitor in <4 min at the approaching airspeed of 15 m s^−1^. Subsequently, an array of FTENGs can be readily created for substantial scale energy farms. Fluttering has some limitations in collecting energy from breezes with random orientations.^38^

To overcome this limitation, a lightweight and freestanding woven TENG (WTENG) design has been proposed, as outlined in Figure 4a(II).^39^ The WTENG banner was woven by conductive belts of Ni‐covered polyester materials (Ni belts) and Kapton film–sandwiched Cu belts (KSC belts). The entire WTENG banner is shape adaptable so that it can collapse or bend and has a weight under 15 g, significantly lighter than other wind‐based devices. It uses commonly available materials, and the entire manufacturing process is economically justifiable for large‐scale deployment. This design can generate an open‐circuit voltage, a short‐circuit current, and a power density reaching 50 V, 70 µA, and 140 mW kg^−2^, respectively, at a wind speed of 14 m s^−1^. An adaptable and straightforward flexible TENG for arbitrary wind energy harvesting has also been proposed. In this design, freestanding polymer strips, vertically oriented, are made of an indium tin oxide (ITO)‐covered polyethylene terephthalate (PET) thin film. The laminar TENG network assumes a kelp forest morphology, and every strip movement generates an autonomous influence to cause contact separation when a breeze passes by.^40^ The freestanding polymer strips operate vertically at a frequency as high as 154 Hz with adequate contact separation. With a strip size of 10 cm × 2 cm under a wind speed of 27 m s^−1^, two nearby strips 2 × 0.7 cm wide can produce an open‐circuit voltage, a short‐circuit current, and a power density reaching 98 V, 16.3 µA, and 2.76 W m^−2^, respectively. Figure 4a(III) shows photos of a TENG from multiple viewpoints, where the device has internal measurements of 100 × 10 × 1.6 mm^3^.^41^ The wind streams into the holes of the device and instigates the vibration of the Kapton film, which can drive the energy harvesting action of the TENG. Occasional contact of and separation between the Cu terminal on the Kapton film and the PTFE film occur under the wind stream. TENG dimensions of 125 × 10 × 1.6 mm^3^ bring about a maximum output power density of ≈9 kW m^−3^ under an external resistance of 2.3 MΩ and generate an open‐circuit voltage and a short‐circuit current of 500 V and 140 µA, respectively.

### 5.2 Rotary‐Based Mechanism

A blow‐driven (BD) TENG can generate energy via exhalation.^44^ The critical structure of this configuration, for the most part, is composed of three functional parts: a rotator, a stator and a delicate flexible material (e.g., a wipe, to act as a spacer), as schematically shown in Figure 4b(I).^42^ The proposed TENG delivers an instantaneous output power of 19 mW (102 W m^−3^) at a load of 0.8 MΩ, an *I*_SC_ of 0.4 mA, and a *V*_OC_ of 450 V.

Figure 4b(II) introduces a structure consisting of TENGs for large‐scale energy harvesting from the natural wind.^19^ In the contact electrification–based freestanding mode between two disks, low and high wind energy is converted into electricity. Moreover, one unit can produce an open‐circuit voltage, a short‐circuit current, and an output power of 600 V, 0.5 mA, and 0.25 W at a load of 5 MΩ. Finally, the performances of TENGs and Darrieus turbines are compared both experimentally and theoretically, predicting the unique advantage offered by a TENG at low rotation speed.^19^ A rotary TENG (R‐TENG) was designed with the intent of harvesting energy from low‐amplitude wind in the surrounding media, as depicted in Figure 4b(III).^43^ The power production of the R‐TENG results from combined contact and sliding separation, with the cycle closed upon contact. Based on this new design, a *V*_OC_ of 250 V and an *I*_SC_ of 0.25 mA have been achieved, with an extreme power yield of 62.5 mW.

## 6 Hybrid Nanogenerators

Both wind and hydrokinetic energy harvesting can benefit from TENGs assuming their multifaceted abilities.^46^ This section will introduce some of the latest innovations in the field of hybrid energy harvesting, including TENGs, electromagnetic generators (EMGs), and solar cells (SCs).

### 6.1 TENG‐EMG Hybrid Generator

**Figure 5:**
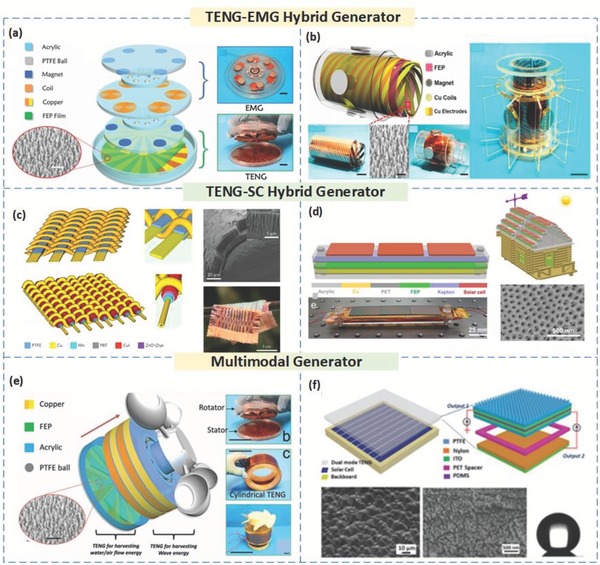
Hybrid and Multimodal‐based TENGs for Scavenging Blue Energy. a) The structure design and an actual photo of a hybrid energy harvesting system (TENG and EMG). Reproduced with permission.^47^ Copyright 2016, John Wiley & Sons. b) Schematic illustration and a real photo of a hybrid nanogenerator (S‐TENG and W‐EMG). Reproduced with permission.^12^ Copyright 2016, American Chemical Society. c) Schematic illustration and an actual photo of a hybrid power textile. Reproduced with permission.^48^ Copyright 2016, Nature Publishing Group. d) Schematic diagram and a real photo of an integrated hybridized nanogenerator. Reproduced with permission.^50^ Copyright 2016, American Chemical Society. e) Structural design and a real photo of a multifunctional TENG, which mainly consists of two parts: a rotational TENG and a vertical cylindrical TENG. Reproduced with permission.^51^ Copyright 2017, John Wiley & Sons. f) Schematic diagram and a real photo of an as‐fabricated hybridized power panel. Reproduced with permission.^52^ Copyright 2015, John Wiley & Sons.

Here, we mention a waterproof triboelectric (TENG)–electromagnetic (EMG) hybrid generator (WPHG) for energy collection under more elaborate conditions.^47^ Since the transmission of mechanical energy from the external mechanical source to the TENG is through a noncontact force between paired magnets, an entirely isolated packaging of the TENG part can be easily achieved. At the same time, by combining these magnets with metal coils, an EMG can be fabricated. The device structure of the multilayered WPHG is schematically outlined in **Figure** **5**a. The TENG and EMG produce output powers of 1 mW at 1 kΩ and 1.5 mW at 1.2 kΩ, respectively. By using transformers and full‐wave rectifiers, a 2.3 mA total short‐circuit current, and a 5 V open‐circuit voltage are obtained for the WPHG under a rotation speed of 1600 rpm, which can charge a supercapacitor (20 mF) to 1 V in 22 s. Another system that combines a winding interdigital‐electrode TENG (S‐TENG) and a wrap‐around electromagnetic generator (W‐EMG) was utilized for water wave energy harvesting.^12^ Its performance indicates that an S‐TENG has a better affinity for harnessing hydrokinetic energy at low frequencies than a W‐EMG with a more extensive frequency domain. The fundamental structure of the hybrid nanogenerator comprises two principal parts, the S‐TENG, and W‐EMG, which were manufactured from three coaxial tubes, as shown schematically in Figure 5b. The S‐TENG and W‐EMG produced output powers of 25 and 6 µW m^−2^, respectively.

### 6.2 SC‐TENG Hybrid Generator

Here, we introduce a hybrid energy harvesting and storage system which can be considered as a self‐powered charging cell.^48^, ^49^, ^50^ The primary framework for the design is to utilize a polymer fiber–based SC as the essential segment in creating a TENG so that both sun‐based and mechanical energy sources can be harvested at the same time. The wearable hybrid energy fabric has an interwoven structure of separate layers, which consists of a blend of two polymer wire–based energy devices, including both a textured TENG to convert mechanical energy into power and a photovoltaic material to harvest power from exposure to sunlight, as schematically depicted in Figure 5c. The hybrid power textile with a size of 4 cm by 5 cm is able to stably deliver an output power of 0.5 mW over a huge range of resistance from 10 kΩ to 10 MΩ.^48^ Additionally, other structural designs have been recently described in the form of a hybridized nanogenerator that comprises an SC and a TENG, which can be used to exclusively/concurrently harness sunlight and wind energies.^50^ In Figure 5d, with an area of ≈120 mm × 22 mm, the SC can generate an energy yield of 8 mW in the current configuration. The energy yield of the TENG can be up to 26 mW. Extensive scale deployments of the hybridized nanogenerators on city roofs can expand solar and wind energy scavenging in urban districts to provide a certain degree of self‐fueled capacity within the context of a smart city. Moreover, the measured output voltage and current signals of the SC are ≈7 V and 9 mA under full‐sun intensity (100 mW cm^−2^). The open circuit voltage and short circuit current of the TENG are 600 V and 300 A, respectively. The hybridized nanogenerator has a larger output current and a better charging performance than the individual SC or TENG.^50^

## 7 Multimodal TENGs

Multimodal energy systems can be realized as an additional feature to be integrated with other energy harvesting technologies using TENGs. This section will discuss some of the latest works in the field of multimodal functionalities of blue energy TENGs. A multifunctional TENG that can be employed in the ebb and flow of oceanic tides is shown in Figure 5e, which is fit for synchronous harvesting of wave, water flow, and wind energies.^51^ This TENG comprises, for the most part, two sections: a rotational part and a vertical tube–shaped part. The rotational TENG (r‐TENG) is utilized to simultaneously collect wind and water flow energy. The working system of the multifunctional TENG depends on coupled triboelectric impact and electrostatic induction.^51^ For the r‐TENG, which is utilized to scavenge wind and water flow energies, the working component depends on the freestanding mode of TENG operation, which includes three noteworthy stages. The impact and freestanding modes have output voltages of 490 and ≈100 V with short‐circuit currents of 24 and 2.7 µA, respectively when operated at a rotation frequency of 200 rpm. Moreover, the multifunctional TENG can produce an output power of 0.15 mW. On a breezy day, a hybridized cell can convey a nominal yield of 8 mW m^−2^ at a wind velocity of 2.7 m s^−1^.^52^ For a similar configuration on a stormy day, energy collection from dripping water drops attains a nominal yield of 86 mW m^−2^ at a dripping rate of 13.6 mL s^−1^. The introduced hybridized cell is a novel and simplistic design toward a high‐productivity environmentally friendly energy resource. The hybridized power board, for the most part, comprises three sections: an acrylic backboard, an SC made from silicon, and a double mode TENG, as schematically shown in Figure 5f.

## 8 Large‐Scale Energy Harvesting Based on TENGs

Though large‐scale energy harvesting is mainly dominated by energy farms and plants, WSNs and IoT applications can be powered by other means. Other solutions apart from TENGs include photovoltaic cells and electrostatic, piezoelectric, and thermoelectric generators. However, other solutions generally involve traditional energy plants and renewable energy plants. Up to now, we have highlighted some of the significant advantages of using a TENG as an energy harvester. These advantages include the ease of fabrication, lightweight, scalability, and shape adaptivity. These features make TENGs a strong candidate for large‐scale energy plants for sustainable energy harvesting to operate WSNs, smart city electronics, and IoT applications. For example, installing a vast TENG network in the ocean has been suggested as a possible solution for harvesting water wave energy.^24^ This approach may provide a low‐cost solution for harvesting ocean water wave energy. Considerations for such a network include but are not limited to cable connections, durability under harsh environments, and plastic pollution. Here, we highlight the significant achievements in the field of large‐scale energy harvesting and IoT devices operated by TENG‐based technologies.

### 8.1 Water Wave Networks Based on TENGs

**Figure 6:**
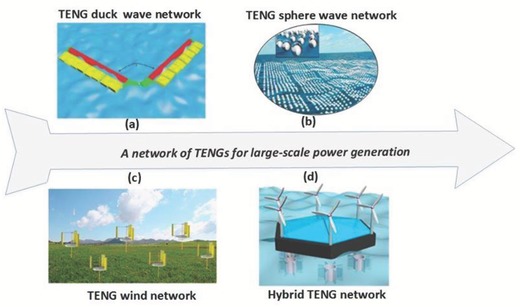
A network of TENGs for large‐scale power generation. a) A V‐shaped network of duck units based on a WEPTOS WEC model for wave energy harvesting. Reproduced with permission.^5^ Copyright 2017, The Royal Society of Chemistry. b) A TENG network composed of millions of spherical balls for harvesting large‐scale blue energy. Reproduced with permission.^3^ Copyright 2017, Elsevier. c) Schematic illustration of the configuration for wind energy harvesting using a TENG farm. Reproduced with permission.^19^ Copyright 2017, Elsevier. d) Schematic illustration of an energy harvesting panel floating on the ocean, which mainly consists of wind‐driven generators, solar cell panels, and arrays of hybrid nanogenerators. Reproduced with permission.^12^ Copyright 2016, American Chemical Society.

A probable approach to harvesting blue energies is the use of networks of TENGs that can collectively boost the amount of harvested energy. For instance, in a unique design, three same duck shape TENG units, each containing four layers, were developed. Considering a hybridized framework, the units were linked in parallel and examined under the ideal frequency range ascertained earlier. Accordingly, the resistance reliant on the produced electrical power was measured for various numbers of units, *n* = 1, 2, 3, as depicted in **Figure** **6**a.^5^ The maximum power improved as the number of units increased, and the highest maximum power for *n* = 3 was determined to be 1.366 W m^−2^. A conceivable system can be proposed with the WEPTOS WEC configuration, as indicated in Figure 6a. Further progress can be made with the duck units by applying them to the two legs of a floating structure in light of the first WEPTOS WEC setup.^5^ In this manner, the direction of the loaded units can be efficiently changed by incident waves, keeping in mind the end goal of collecting the optimum energy levels from the water wave flow. This edge distance variability can additionally enable the devices to interface significantly less with waves in heavy loading conditions to moderate such elevated applied forces. In another example, Figure 6b shows a TENG network composed of several spherical ball–based TENG units.^3^ These sphere‐shaped TENGs have lightweight organic and metal parts and are partially occupied by air so that they can float on the water surface.

### 8.2 Wind Farms Based on TENGs

Figure 6c shows the schematic of a TENG farm design. Here, a variety of TENG breeze harvesters in an electrical network are proposed.^19^ The separation between every unit is twice the TENG's harvester elevation. For such a system, an estimation of the power was conducted for 2 km^2^ of TENG wind collectors and 2 km^2^ of breeze turbines. For a locale with ω < 35 rad s^−1^, the TENG breeze collectors produce a higher yield than the breeze turbines. This configuration may be installed on buildings rooftops so that, through integration with appropriate electrical systems, enhancement in yield performance can be achieved.^19^ Comparing current advances in wind energy technologies, TENG farms hold exceptional advantages for substantial scaling up of wind energy collection. Thus, a TENG farm is considered to be a useful choice to collect breeze energy at an extensive variety of wind velocities, particularly at low speeds. Additionally, since TENGs are generally produced using polymer materials without magnets or substantial amounts of metal, the heap load of the overall device is expected to be diminished mainly compared to current breeze turbines, which are often produced using bulky and expensive materials (for example, metals). Owing to its lightweight, construction of a TENG network with a height of 14 m is reasonable; then, the generated power for a 2 km^2^ network area could be predicted to be 1.11 MW. Moreover, the hybridized nanogenerator gives an altogether promising system to harness sea ebb and flow, tidal, and wave energies from low to high‐frequency ranges. Moreover, we highlight an energy harvesting board panel floating on the sea, which comprises wind‐driven generators, sun‐based cell panels, and a variety of nanogenerators, as schematically outlined in Figure 6d.^12^

## 9 IoT Applications Enabled by TENGs

**Figure 7:**
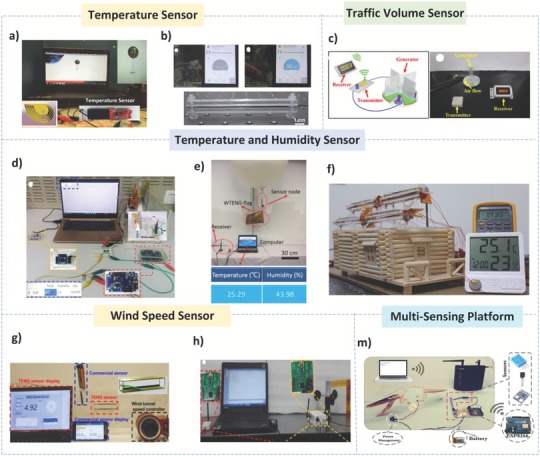
IoT applications based on TENGs. Temperature sensors. a) Demonstration of the wireless temperature sensor node enabled by a duck shape TENG. Reproduced with permission.^5^ Copyright 2017, John Wiley & Sons. b) Demo of a wireless smart temperature sensor powered by a TENG. Reproduced with permission.^53^ Copyright 2016, American Chemical Society. Traffic volume sensor. c) Demonstration of a wireless traffic volume sensing system powered by a hybridized nanogenerator. Reproduced with permission.^54^ Copyright 2016, American Chemical Society. Temperature and humidity sensors. d) Schematic illustration of a self‐powered wireless weather station enabled by a rotary TENG for measuring temperature and humidity. Reproduced with permission.^55^ Copyright 2017, John Wiley & Sons. e) Illustration of powering a wireless temperature and humidity sensor node by harvesting high‐altitude wind energy. Reproduced with permission.^39^ Copyright 2016, American Chemical Society. f) Representation of a temperature‐humidity sensor node powered by a hybridized nanogenerator. Reproduced with permission.^50^ Copyright 2016, American Chemical Society. Wind speed sensors. g) Demonstration of real‐time airspeed measurement using an AF‐TENG sensor and a commercial sensor. Reproduced with permission.^56^ Copyright 2017, Elsevier. h) Diagram of a self‐powered, remote meteorological monitoring system enabled by a rotary wind‐based TENG. Reproduced with permission.^57^ Copyright 2016, American Chemical Society. Multisensing platform. m) Real photo of a self‐powered wireless environmental sensor node (pressure–temperature–humidity) enabled by an H‐TENG. Reproduced with permission.^4^ Copyright 2017, Nature Publishing Group.

The global market for IoT has witnessed substantial growth in recent years. This trend, along with the ever‐decreasing cost of electronics and networking, has led to significant development in the field of communication. **Figure** **7** highlights some of the IoT applications that can be enabled by using a network of TENGs. To demonstrate their scalable design and unique self‐controlled working capacity, three duck‐based TENGs were electrically linked in parallel for 60 min, under a frequency of 2.5 Hz.^5^ This setup was used to run a temperature sensor node (required input power above 20 mW for 20 ms) for 35 s after charging a capacitor (1 mF) and display the temperature value on the monitor, as shown in Figure 7a.^5^ In addition, a self‐powered system consisting of a TENG, a wireless smart temperature sensor node, and an iPhone for receiving the temperature data is shown in Figure 7b.^53^ The 10 mF capacitor was charged in 98 s up to 3.3 V, which fluctuated after turning on the temperature sensor node. At a distance of 26 m, the self‐powered wireless smart temperature sensor node can send the temperature data to the iPhone, as shown in Figure 7b.^53^ Figure 7c shows the self‐powered sensing system of a rotating disk–based hybridized electromagnetic TENG based on wind energy from a passing vehicle.^54^ This self‐powered wireless traffic volume sensor will meaningfully aid in real‐time traffic volume tracking at a vehicle speed of 8 m s^−1^, which will offer exceptional convenience, especially in remote mountain areas.^54^

To show the ability to use hybrid NGs in IoT applications, Figure 7d displays an actual photo of a fabricated self‐powered wireless temperature and humidity sensing system, which consists of a hybrid NG, a simple power management system, a sensing system, and a wireless signal transceiver module.^55^ Activated by fluctuating water and wind sources, the hybrid NG can activate the temperature and humidity sensor after charging a lithium‐ion battery (10 mAh) using the hybrid NG in 270 s.^55^ Furthermore, a self‐powered high‐altitude platform (HAP) with wireless temperature and humidity sensors forms a meteorological node, whose required voltage and current are ≈3.5 V and 10 µA, respectively, as shown in Figure 7e.^39^ The battery with the WTENG flag was charged at a wind speed of 14 m s^−1^ for 4.8 h and later displayed a sufficient voltage of ≈3.5 V and a discharge capacity of 10 µAh at a release current of 1 µA.^39^ A good system for a smart city is illustrated in Figure 7f, which shows a demo of hybridized nanogenerators integrated by installing them on the rooftops of city houses. Four devices were linked in parallel and installed on the rooftop of a building model, as demonstrated in Figure 7f.^50^ One TENG can generate an output current of ≈4.5 mA, while the four combined hybridized nanogenerators simultaneously produce a total output current of ≈10.5 mA when harvesting solar and wind energies in several seconds. Then, the temperature–humidity sensor can be driven using a lithium‐ion battery (with a 0.8 V onset voltage) that can be charged by the hybridized nanogenerators, as demonstrated in Figure 7f.^50^

Furthermore, the flutter‐based TENG “AF‐TENG” has been used as a wind speed sensor (Figure 7g).^56^ To further demonstrate the AF‐TENG performance, a comparison between the real‐time speeds from the AF‐TENG and a commercial hot‐wire anemometer was performed. In addition, Figure 7h displays an actual image of an established self‐powered wireless remote weather monitoring system including a TENG, a transmitter and a receiver connected with a computer.^57^ The TENG system can energize the wind sensor and transmitter by using wind energy and charging the battery in two modes. The first mode is the standby mode, in which the battery is charged to 3.16 V for 14.7 h, while in the active mode, the battery releases a 2 µA current for 2.4 h. Consequently, the established self‐powered wireless wind speed sensing system can be used for remote meteorological monitoring, which provides great convenience for real‐time weather and environmental observation.^57^ The constructed bioinspired H‐TENG (Figure 7m) can be used as a stable power supply for IoT applications to operate multisensing platforms for measuring temperature, humidity, and atmospheric pressure, as shown in Figure 7d.^4^ Six H‐TENG devices were connected in parallel under a wind speed of 7.5 m s^−1^ for 60 min to provide power to a wireless sensing module for receiving and sensing signals at 1 s intervals, which was operated using the energy stored in a battery with a capacity of 70 mAh and a battery charging module, as illustrated in Figure 7m.^4^

## 10 Self‐Charging Power Units Based on TENGs

Research efforts to improve the performance of TENGs have reached power density levels in the range of 500 W m^−2^, with an instantaneous conversion efficiency of 85%. This yield is sufficient to successfully deliver the power demand for many portable devices. However, unaddressed challenges still need to be resolved before TENG‐based devices can be commercially available. First, being able to secure stable power from a TENG is crucially essential considering that the dependency of such a device on unpredictable parameters, such as weather, can be problematic.^58^ The same issue applies to solar, wind, and any other energy sources with an intermittent availability nature. Additionally, the fact that TENG‐based devices produce power with changing frequencies (i.e., AC power) makes their direct use in many types of electronic devices extremely challenging. This issue leads to consideration of a device that, in addition to harvesting energy, can effectively store energy so that the constant DC power required by most electronic devices can be provided.^59^ In this regard, SCPUs have received much attention, which is realized by pairing TENGs with energy storage devices such as batteries and capacitors.^60^ However, direct integration of a TENG with an energy storage device leads to substantial energy losses because of the significant impedance mismatch between the two devices.^61^ A TENG usually has output characteristics of a high voltage, a low current and a sizable internal impedance (≈10^6^ Ω) several orders of magnitude higher than that of batteries. Therefore, developing a proper power management strategy to achieve a proper impedance match between the energy‐generating TENG and energy‐storage cells is essential to maximize the total efficiency of the system.

### 10.1 Design of the Power Management Circuit

#### 10.1.1 Direct Integration

**Figure 8:**
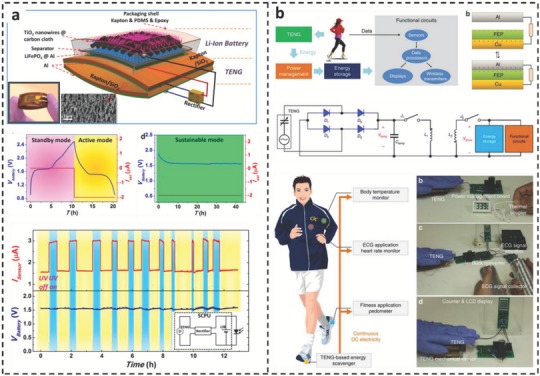
Structure and performance of different self‐charging power units (SCPUs). a) The structural design of a flexible SCPU, performance of the SCPU as an integrated DC power source, and the voltage profile showing the charge and discharge characteristics of the lithium‐ion battery storage element. The SCPU provides a 2 µA DC current with a constant voltage of 1.53 V for more than 40 h. The operation of a UV sensor continuously driven by the SCPU in the “sustainable mode” for ≈13 h. Reproduced with permission.^62^ Copyright 2013, American Chemical Society. b) System diagram of a triboelectric nanogenerator (TENG)‐based self‐powered system, working mechanism of an attached‐electrode contact‐mode TENG, a circuit diagram of the power management circuit, and system configuration of self‐powered human activity sensors for temperature and heart rate monitoring and a pedometer. Reproduced with permission.^59^ Copyright 2015, Nature Publishing Group.

The most challenging part of designing a power management circuit is to find the optimal path through which the generated power and storage efficiency would be maximized. In a power management circuit with the simplest design, known as direct charging or direct integration, the AC signal produced by the TENG device is converted into DC voltages through a bridge rectifier. This conversion then enables direct storage of the harvested energy in a large capacitor or battery.^62^ Wang and coworkers demonstrated the first flexible SCPU capable of simultaneously harvesting and storing ambient mechanical energy following a direct charging approach (**Figure** **8**a).^62^ This capability was achieved by integrating a flexible lithium‐ion battery with a mechanical energy harvesting TENG in one device, as schematically illustrated in Figure 8a. In this device, an arch‐shaped TENG generates electricity from ambient mechanical vibrations, and the rectified current is used to fully charge the lithium‐ion battery in 11 h. This sustainable SCPU can provide a continuous DC current of 3 µA at a constant voltage of 1.55 V and is capable of continuously powering a UV sensor. While this design has many advantages, such as simple integration, it suffers from very low charging efficiency due to the very large impedance mismatch between the energy harvesting and storage devices.^63^ Calculations show that if a TENG is used to charge an ideal 1.0 V battery directly, the theoretical charging efficiency is only 1.1%, even when the battery's leakage current and internal resistance are neglected.^59^ Enormous power is lost during the charging process, leading to a few microwatts of DC power, which is insufficient for most practical applications. Recently, a spring‐assisted multilayered structure integrated with a power management module (PMM) was constructed to harvest water wave energy. Using this integrated system, the stored energy for charging a capacitor is intensely enhanced by up to 96 times.^64^

#### 10.1.2 Transformer Integration

In another approach, the impedance can be matched between the battery and its corresponding TENG through a transformer. Using this method, with the aim of significantly improving the output performance of TENGs, Zhu et al. proposed an effective energy conversion system based on the stator–rotator structure with arrays of microsized radial segments.^30^ This planar‐structured TENG produces a variable triboelectric potential that induces an AC between the electrodes. When operating at a rotation rate of 3000 rpm, the measured open‐circuit voltage of the TENG can reach 850 V, leading to a continuous short‐circuit current of 3 mA at a constant frequency of 3 kHz. This system suffers from a large impedance, which limits its applicability as a power source. To address this issue, the authors integrated the TENG with a power management circuit consisting of a transformer, a rectifier, a voltage regulator, and capacitors. As a result, the transformer was able to significantly reduce the impedance by boosting the output current and lowering the operating voltage. The new system was then able to deliver a DC output at a constant voltage of 5 V only 0.5 s after operation of the TENG. This TENG can deliver an output power of 1.5 W (power density of 19 mW cm^−2^) at the matched load of 0.8 MΩ, with an improved efficiency of 24%, which represents a significant leap forward compared with previously reported power management systems based on rectifiers.^43^ This system has the capability of working at relatively low frequencies in the range of up to 3000 Hz, which, for many applications, is not ideal. Historically, transformers have been used as the primary power management element for energy harvesters, but they lead to appreciable power loss in the case of TENGs.^54^, ^65^ This loss can be attributed to the fact that transformers work best at designed frequency and bandwidth, which cannot be easily satisfied by TENGs that mainly harvest low‐frequency energy with random pulsed power.^66^

#### 10.1.3 Power Management Circuit Board

A configuration consisting of a TENG, a low‐leakage energy storage device, and a power management circuit was used to show the first effective self‐powered system capable of delivering the power demands of personal electronics.^59^ The authors carried out system‐level optimization to ensure that all the system components work together cooperatively. A unique circuit was custom designed to reduce the impedance mismatch, leading to a 90% board efficiency and a 60% total efficiency, approximately two orders of magnitude improvement compared to direct charging. This excellent performance was achieved via a two‐stage power management circuit, as schematically illustrated in Figure 8b. First, a temporary capacitor is charged by a TENG through a bridge rectifier. Second, two electronic switches drive the transfer of this energy to a larger capacitor to maximize the total charging efficiency. To demonstrate its applicability, the authors used this system to convert widely available biomechanical energy to sustainably drive a broad range of commercial cellphones and wearable electronics, as shown in Figure 8b. This power unit provides a continuous DC electricity of 1.044 mW (7.34 W m^−3^) in average power in a regulated manner.^59^

### 10.2 Energy Conversion Efficiency

As we have discussed, overcoming the impedance mismatch is crucial, and several efforts have been made to develop power management circuits that can successfully lower or eliminate the impedance mismatch and therefore improve the overall charge storage efficiency. **Table** **3** shows the performance of SCPUs with various power management strategies based on switched capacitors,^67^ oscillating inductor‐capacitor (LC) circuits,^68^ high‐voltage diodes,^69^ rectification storage, and DC–DC management circuits,^69^ specially designed charge cycles,^71^ etc. While excellent energy transfer efficiencies on the order of up to 80% have been achieved, further research is still needed to ensure high efficiency. Currently, integrated energy harvesters and energy storage devices can possibly replace batteries or at least extend the lifetime of batteries. To decrease the energy conversion loss, self‐charging power cells can provide an effective solution by hybridizing an energy harvester with an energy storage unit. Moreover, such integrated systems require energy storage devices with high energy in a small volume and low weight to realize longer lifetimes and less repeated recharging. Additionally, an energy storage system can steady the output of the energy harvesting system when the load varies rapidly and meaningfully. Integrated systems are a feasible solution to solve dynamic power quality problems, such as voltage sag. For this technology to reach the market, smaller, lighter, low‐cost materials, and more easily integrated power management units for large‐scale production are still required.

**Table 3:** Comparison of different power management circuits aimed at reducing the impedance mismatch between energy harvesting and storage devices. The goal is to increase the energy transfer efficiency, which is defined as the ratio of the power that can be stored in the storage element to the maximum AC power that can be harvested by a resistive load

Rectifiers	Lithium‐ion battery, 1.53 V	Contact‐separation mode (CS)	3 µW	NA	1.1%	62
Transformers	NA	Freestanding mode (FS)	1.5 W @ 0.8 MΩ	NA	24%	30
Switched capacitors	Supercapacitor, 5 mF	Sliding‐freestanding (SFT) mode	30.3 µW @ 350 MΩ	Energy transfer increased by 19.64 times	23%, 57.3%	67
Inductor‐capacitor (LC) oscillating circuit	Capacitor, 1 µF to 4.7 mF	Lateral‐sliding (LS), contact‐separation (CS) mode	67.6 µW @ 100 kΩ	Over 2600 times improvement in stored energy	72%	68
High‐voltage diode	Capacitors 1 nF, 10 nF, 1 µF Supercapacitors 1, 10 µF	Contact‐separation mode (CS)	NA	NA	NA	69
Inductor‐capacitor LC circuits	Capacitor, 1 mF	Sliding‐freestanding, contact‐separation (CS) mode	11.2 µW@ 35 MΩ	Stored energy improved by 128 times	80%	70
Rectification storage circuit and DC–DC management circuit	Capacitor, 10 mF	Freestanding (FS) mode	106 mW @ 166 kΩ	Stored energy improved by 15 times	50%	69
Bridge rectifiers, capacitors	Capacitor, 1 mF	Attached‐electrode contact mode	1.044 mW (7.34 W m^−3^) 0.3384 mW @ 4.25 MΩ	NA	59.8%	59
Bridge rectifier, motion‐triggered switch for charging cycle	Capacitor 0.73 µF, Lithium‐ion battery 3.8 V	Sliding‐freestanding (SFT) mode	28.8 µW	NA	50%	71
Capacitors, charging chip, rectifying circuit, comparator chip, and switch chip	Lithium‐ion battery	Contact‐separation (CS), lateral‐sliding (LS) mode	NA	Stored energy improved by 1.75 times	NA	72
Transformer, rectifier, and low‐leakage capacitor	Capacitor, 10 µF	Freestanding (FS) mode	2.28 mW @ 80 MΩ	NA	57.9%	72

### 10.3 Design of a Supercapacitor and Battery Integrated TENG

#### 10.3.1 Batteries and Supercapacitors for Small Electronics

**Figure 9:**
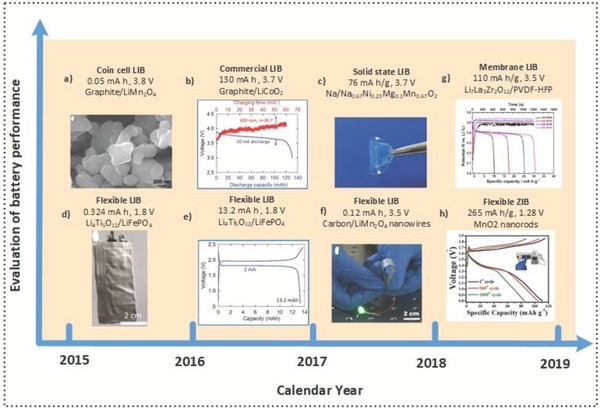
Timeline of batteries for self‐charging power units. a) A coin cell lithium‐ion battery (LIB). Reproduced with permission.^73^ Copyright 2016, Nature Publishing Group. b) A commercial LIB. Reproduced with permission.^74^ Copyright 2016, John Wiley & Sons. c) A solid‐state LIB. Reproduced with permission.^75^ Copyright 2017, John Wiley & Sons. d) A flexible LIB. Reproduced with permission.^76^ Copyright 2015, John Wiley & Sons. e) A flexible LIB. Reproduced with permission.^74^ Copyright 2016, John Wiley & Sons. f) A flexible LIB. Reproduced with permission.^77^ Copyright 2017, John Wiley & Sons. g) A triboelectric nanogenerator (TENG) in an all solid–state LIB. Reproduced with permission.^78^ Copyright 2018, Elsevier. h) A triboelectric nanogenerator and a zinc‐ion battery on designed flexible 3D spacer fabric. Reproduced with permission.^79^ Copyright 2018, John Wiley & Sons.

The energy storage component is an integral part of self‐charging power systems. To create such systems, the properties of energy storage devices must meet some requirements that we will discuss in the next sections. One of the primary goals of energy research is to provide a sustainable power source for powering portable and wearable electronics purely by harvesting biomechanical energy from daily motion. If the average working time of a human body is 6 h per day, this movement could produce 3.4 Wh of energy, which is sufficient to fully charge most of today's smartphone batteries. While TENGs have shown great capability for harvesting human motion, without efficient energy storage, producing a sustainable power source will be impossible. Over the last three years, we have seen some developments in the fabrication of energy storage devices that can be integrated into wearable, self‐charging electronics.^80^ These devices include flexible batteries,^74^, ^76^, ^77^ round coin cells,^73^ and even safer solid‐state batteries,^75^ as illustrated in **Figure** **9**. However, the capacity is well below 15 mAh, which is too small for most commercial portable and wearable electronics. For these systems to become practical, the development and integration of batteries with a capacity of ≈1000 mAh, just enough to run small portable electronics, are necessary.

For instance, in 2017, a highly stable (capacity retention of 90% after 50 cycles) and efficient flexible lithium‐ion battery was introduced that was constructed from electrospun LiMn_2_O_4_ nanowires as the cathode and carbon nanowires as the anode. This battery can reach 3.5 V in the charging sequence within 3 min under wind‐induced fluctuations.^77^ In 2018, a solid‐state lithium battery with a hybrid solid electrolyte (HSE) membrane was fabricated. Consisting of a metal anode and a LiFePO_4_ cathode, this battery can deliver 110 mAh g^−1^ after 180 cycles at a 0.5 C rate.^78^

Another approach for a flexible LIB exploited the developing LiMn_0.6_Fe_0.4_PO_4_/carbon (LMFP/C) material within a self‐charging power system. This battery can reach a specific capacity of 90 mAh g^−1^ at a current density of 1 C, which is approximately five times greater than that of LiMnPO_4_/C, and it maintained the same performance after 1000 cycles and exhibited no degradation after 300 bending cycles.^81^ Recently, a flexible 3D spacer design was introduced by integrating a TENG and a rechargeable zinc‐ion battery (ZIB). Regarding its distinctive shape, the flexible ZIB can obtain a specific capacity of ≈265 mAh g^−1^ at a current rate of 1 C and cyclic stability over 1000 cycles (76.9% capacity retention). Moreover, the charging voltage increased from 0.93 to 1.28 V when using the integrated system, which has a discharge capacity of 10.9 µAh at a 4 µA current density, which can be used to power an electronic watch.^79^

**Figure 10:**
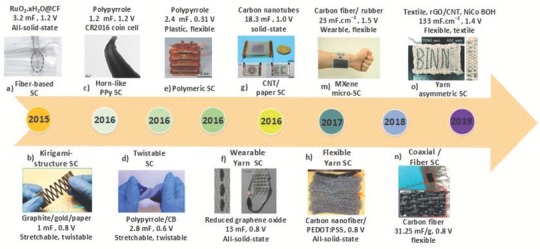
Timeline of essential milestones in supercapacitors for self‐charging power units. a) A fiber‐based solar cell (SC). Reproduced with permission.^84^ Copyright 2015, John Wiley & Sons. b) Kirigami structure. Reproduced with permission.^85^ Copyright 2016, American Chemical Society. c) Horn‐like Ppy SC. Reproduced with permission.^73^ Copyright 2016, Nature Publishing Group. d) Twistable SC. Reproduced with permission.^85^ Copyright 2016, American Chemical Society. e) Polymeric SC. Reproduced with permission.^86^ Copyright 2016, John Wiley & Sons. f) Wearable yarn. Reproduced with permission.^74^ Copyright 2016, John Wiley & Sons. g) CNT/paper SC. Reproduced with permission.^61^ Copyright 2016, The Royal Society of Chemistry. h) Stretchable yarn SC. Reproduced with permission.^83^ Copyright 2017, American Chemical Society. m) MXene electrochemical microsupercapacitors integrated with a triboelectric nanogenerator. Reproduced with permission.^87^ Copyright 2018, Springer. n) Coaxial TENG and fiber SC. Reproduced with permission.^88^ Copyright 2018, American Chemical Society. o) All yarn‐based energy harvesting triboelectric nanogenerator. Reproduced with permission.^89^ Copyright 2019, John Wiley & Sons.

Supercapacitors are alternative energy storage devices that have attracted attention over the past two decades because of their high power density and excellent cycle life compared with batteries.^82^ Given the alternating nature of the energy generated by TENGs, supercapacitors may provide a better means for storing this energy. Additionally, the long cycle life of supercapacitors is desirable for devices that require a sustainable and maintenance‐free power source, such as sensors and health monitoring devices. **Figure** **10** summarizes some recent work on the integration of supercapacitors and TENGs. A self‐charging power system that seamlessly integrates a stretchable TENG with a stretchable supercapacitor in a single package has been developed.^83^ (Figure 10h) The entire device is made of soft materials that can be not only bent but also stretched, twisted, and even shaped into complex structures, and all such deformations can be converted into electricity. By mounting this device onto a human body, the authors showed the capability of the device to drive an electronic watch. The device is also washable and waterproof due to its fully enclosed structure and the hydrophobic nature of the packaging materials. Striving to improve the capacitance of this device (only 2.8 mF), researchers have demonstrated supercapacitors with capacitances up to 18.3 mF and an excellent cycle life between 5000 and 10 000 cycles^61^, ^73^, ^74^, ^83^, ^84^, ^85^, ^86^ Future research should focus on improving the capacitance, internal resistance, and cycle life of supercapacitors. Additionally, the TENG and the supercapacitor are often two separate components, which limits their functionality. Therefore, integrating these devices into single and durable power units to reduce wiring and electrical contacts and improve efficiency is highly desirable.

Recently, in 2018, an all‐in‐one fiber‐based coaxial device combining a TENG outside and a SC inside was demonstrated. The authors used carbon fiber as the electrode and active material as well for the SC. However, silicone rubber was used to separate the TENG and SC and worked as an active tribo material. The coaxial device has a diameter of 2 mm, and the measured capacitance of the SC is 31.25 mF g^−1^.^88^ Due to its flexibility and high stability, this device can be used for self‐charging textile systems.^88^ Another promising approach has been reported using MXene‐based microsupercapacitors integrated with TENGs. The MXene microsupercapacitors can obtain a capacitance of 23 mF cm^−2^, with 95% capacitance retention after 10 000 charge–discharge cycles. Given the uncomplicated and compact shape, several demonstrations of the self‐charging power band were given to show possible sensory applications^87^ (Figure 10m).

Furthermore, an ultrathin supercapacitor (thickness of ≈170 µm) using a paper sheet and a solid electrolyte in a sandwich design was introduced for conserving the energy generated by a TENG. The hybridized ultrathin SC and flexible TENG were used to detect both static and dynamic pressures.^90^ In 2019, a self‐charging interwoven power textile based on TENGs and yarn‐based asymmetric supercapacitors (Y‐ASCs) was reported, (Figure 10o).^89^ The reported Y‐ASC fabricated with a negative electrode (rGO/CNT coating), and a positive electrode (NiCo BOH) provides the advantages of high‐power output and mechanical stability. CNT fillers were used to separate rGO sheets and form pathways for electrolyte penetration and ion diffusion. Due to the porous morphology and good contact with Cu substrates, rGO/CNT and NiCo BOH electrodes achieve high current rates. Hence, the Y‐ASC obtains a high areal energy density (≈78.1 µWh cm^−2^), a high‐power density (14 mW cm^−2^), stable cycling performance (82.7% for 5000 cycles), and excellent flexibility (1000 bending cycles of 180°). The areal capacitance obtained from the CV curve at 5 mV s^−1^ is ≈133 mF cm^−2^, which is higher than and comparable with those previously reported for flexible SCs.^89^

#### 10.3.2 Large‐Scale Energy Storage

**Figure 11:**
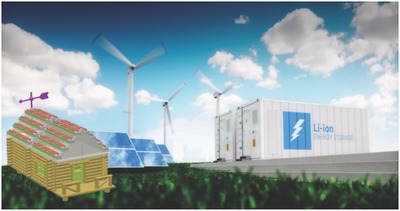
Conceptual design for large‐scale renewable energy harvesting using photovoltaics, wind turbines, and triboelectric nanogenerators. For this system to function appropriately, large‐scale batteries are necessary, as they offer a well‐established approach for improving grid reliability and utilization. The schematic diagram of the nanogenerators on the roof of a city building is reproduced with permission.^50^ Copyright 2016, American Chemical Society.

Recently, wind and solar farms are one of the most promising sources for clean and renewable energy.^91^ According to a recent report from the International Energy Agency (IEA), renewable energy accounted for two‐thirds of the new power added to the world's grids in 2016. The report also noted that solar power is the fastest‐growing source of new energy worldwide. Here, we propose the integration of nanogenerators as an effective method for harvesting wind and water energy on a large scale. **Figure** **11** illustrates a conceptual design for a green city powered entirely by renewable energy. This city secures its electricity needs by harvesting solar, wind, and water energy using nanogenerators in conjunction with well‐established photovoltaics and wind turbines.

Five cities in the United States are currently powered entirely by solar and wind energy. Integration of nanogenerators into the electric grid would improve the reliability and drive the widespread adoption of renewable energy. These nanogenerators offer a simple, yet effective, strategy for energy scavenging from city environments and may eventually enable the sustainable energy supply in a smart city. Although solar energy can be effectively scavenged with existing photovoltaics, a significant portion of wind energy is wasted since wind farms can only be installed outside the city. Similar to SCs, TENGs can be installed on the roofs of residential buildings to harvest wind and solar energy.^50^ Additionally, they can be used to harvest the energy of ocean waves, which would otherwise be lost.^92^ Wang calculated that the world's energy needs could be met by covering an ocean area the size of the US state of Georgia with a 3D nanogenerator network of devices placed 10 cm apart and stretching 10 m deep beneath the surface.^92^

However, renewable energy comes with built‐in challenges: the sun does not shine at night, and wind does not blow all the time. In addition, water waves exhibit random oscillations, with available power over a range of frequencies. The intermittent nature of renewable energy sources is a limitation for the electric grid that requires a continuous supply of electricity around the clock. Batteries provide a solution to this problem by collecting excess electricity for use in times when the sun may not be shining or the wind not blowing. Consequently, to ensure maximum efficiency, the battery, as an essential element for a sustainable power source based on TENGs, should meet the following criteria:

Capacity: Large‐scale batteries are necessary to realize the dream of a TENG‐based sustainable power system for the grid. A good example comes from Tesla Motors, which has recently built the world's biggest battery in the state of South Australia in response to the country's energy crises. Rated at a 129 MWh capacity, the new battery is the size of an American football field and is capable of powering 30 000 homes. The new battery is based on lithium‐ion chemistry, which demonstrates the highest energy density of any rechargeable battery.

Power: The power density of a battery is another critical parameter and determines the speed with which a battery can be fully recharged. It also controls the amount of energy that can be delivered to the grid within a specified period. High‐power energy storage systems may prove very useful for large‐scale wind and water energy harvesting, where they can reduce the recharge time and increase the efficiency of energy transfer from the TENG to the battery.

Cost: The price of the battery pack is a decisive factor and determines the viability of the entire system. Luckily, battery prices are falling very rapidly, especially for electric vehicles and stationary applications. Data between 2007 and 2014 show that the cost estimates of battery packs decreased at a constant rate of 14% annually.^93^ The numbers changed from the above US $1000 kWh^−1^ in 2007 to ≈US $410 kWh^−1^ in 2014. The study also noted that the cost of battery packs produced by market‐leading battery manufacturers is even lower, US $300 kWh^−1^. This number has now declined to US $250 for the Australian battery.

Cycle life: Although a high specific energy density is a good option, this parameter may not be of great importance for grid‐scale storage, which primarily requires a rapid response, high rate performance, and, most importantly, a long cycle life.^94^ To meet these demands, the development of energy storage devices with ≥10 000 charge/discharge cycles would be necessary and be considered a revolutionary advance in the field.

Safety: For batteries to be used for grid‐scale energy storage, they must be free of any potential safety risks. Large batteries should be stored in a secure, dry, and aerated environment, while temperatures need to be controlled at ≈25 °C.

Environmental impact: Because of environmental regulations, some battery chemistries, such as nickel–cadmium, may be excluded due to the toxic properties of cadmium. New battery technologies should be evaluated to determine whether they might pose environmental problems.

When looking at a balance between the advantages and shortcomings of different battery chemistries, many factors must be considered. The initial cost, capacity, power density, lifetime, safety and environmental impacts all play a role in battery selection. The following batteries are potential candidates:

Lithium‐ion batteries: Introduced by Sony in the early 1990s, lithium‐ion batteries have emerged as the most popular rechargeable batteries. Lithium is the lightest of all metals, has the highest electrochemical potential and demonstrates the highest energy density by weight. Such batteries use lithium intercalation compounds for the positive and negative electrodes. During discharge, lithium ions move from the negative electrode through the electrolyte to the positive electrode, causing electrons to flow through the external circuit to power the load.^95^ Lithium‐ion batteries are ubiquitous in portable electronics due to their high energy density, tiny memory effect, and low self‐discharge. They are also widely used for electric vehicles and stationary grid‐scale applications. In fact, the world's largest stationary batteries are based on lithium‐ion chemistry.

Redox flow batteries: Redox flow batteries represent a class of promising electrochemical energy storage devices. Such batteries consist of two tanks of redox active electrolytes that are circulated through two independent loops separated by a membrane.^96^ When pumped into the reactor, the two solutions create a charge to drive a load. Both organic and inorganic redox electrolytes are being used in redox flow batteries to control the operating voltage and other electrochemical characteristics. Redox flow batteries offer an economical means for storing charge at the grid scale, with power ratings from tens of kW to tens of MW and storage durations of 2–10 h.

Supercapacitors: Supercapacitors are energy storage devices, similar to batteries, but they can be charged a hundred to a thousand times faster. Unlike batteries, in which the charge is stored via redox reactions in the bulk of the electrode materials, supercapacitors store charge on the surface of carbon materials with a high surface area of ≈1000–3000 m^2^ g^−1^.^97^ Their high power density and excellent low‐temperature performance make them the technology of choice for power tools, electric buses, and hybrid electric vehicles. Moreover, supercapacitors can be used for an unlimited number of charge and discharge cycles and may have considerable potential for grid‐scale energy harvesting and storage systems.

## 11 Outlook and Future Research Directions

**Figure 12:**
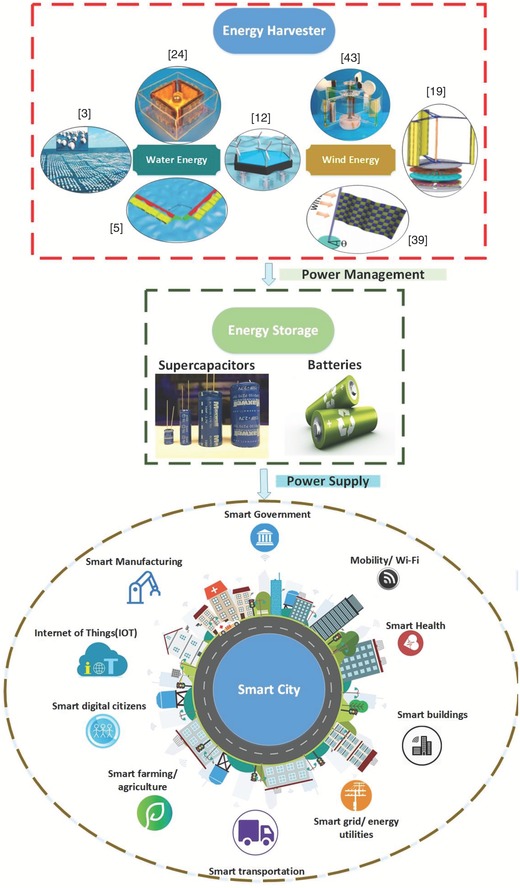
Schematic represents the ideal paradigm of sustainable TENG devices in IoT applications. The first stage would be to provide electronic devices with energy harvesting capabilities. In the second stage, a power management unit with energy storage capacity will ensure optimal functionalities for distributed sensing networks. Finally, a network infrastructure has to be established for linking, monitoring and manipulating functionalities powered by a network of distributed TENG devices for multiple applications within the context of smart city design. Reproduced with permission.^24^ Copyright 2015, American Chemical Society. Reproduced with permission.^5^ Copyright 2017, John Wiley and Sons. Reproduced with permission.^12^ Copyright 2016, American Chemical Society. Reproduced with permission.^3^ Copyright 2017, Elsevier. Reproduced with permission.^39^ Copyright 2016, American Chemical Society. Reproduced with permission.^19^ Copyright 2017, Elsevier. Reproduced with permission.^43^ Copyright 2013, American Chemical Society.

Blue energy in the form of ocean waves offers a tremendous energy resource and can significantly contribute to the energy requirements of our daily life. Due to their low cost, lightweight, easy fabrication, and ability to harvest mechanical energy even at low frequencies, TENGs offer an effective method to harvest the energy from ocean waves. However, several critical challenges remain for blue energy harvesting using TENGs. Since the water from ocean waves can short circuit the TENGs' electrodes, the TENGs must be packaged to isolate contacts. In addition, due to exposure to the harsh marine environment, the packaging has to have specific attributes to be durable: the packaging materials should resist corrosion, protect from the effects of heat and radiation, and preferably be chemically inert. In addition to packaging, energy storage design is essential when considering large networks of TENG devices, with which on‐the‐go functionalities and seamless integration of larger electronics can be established. An example of such distributed networks is self‐powered IoT systems for smart city applications. **Figure** **12** shows the basic design for TENG‐powered devices for sustained IoT operations within a smart city framework. The design illustrates the use of TENGs to harvest energy from water and wind sources available within or around the urban environment. Next, such harvested energy is captured, stored, and managed using energy storage systems and power management units. Energy storage is essential to ensure optimal operation of IoT devices and, quite possibly, extend TENG devices to larger and more distributed network or large‐area electronics. To administer such large networks, the IoT devices must be linked in an information network infrastructure to monitor, secure and possibly manipulate all of the IoT devices with TENG technology in the smart city. The possible applications of such a design paradigm shown in Figure 12 can span many areas, including the medical, transportation, agricultural, manufacturing, and many more industries that require real‐time distributed sensing and data collection. TENGs are still considered to be entirely new compared to other energy technologies, which leaves quite some room for improvement in their already compelling performances. Possible areas for making progress with and enhancing TENGs are suggested below:

1) Establish a standard to characterize the performance of a TENG. Four fundamental TENG modes have been invented, with vastly different structural designs and operating conditions. Therefore, establishing a standard for characterizing the performance of TENGs is critical to perform design optimization, similar to introducing the efficiency and ZT factors for SCs and thermoelectric devices, respectively.^98^ The output power density and energy conversion efficiency are parameters often used to compare the performance of different TENG designs, but the challenge is that these parameters are highly dependent on triggering conditions. A critical factor independent of the triggering conditions might be the charge density on the two triboelectric surfaces.^7^, ^22^, ^71^, ^98^ Quantitative techniques have to be developed to accurately measure the surface charge density to understand how the surface structures, such as the roughness, dielectric properties, and presence of nanoparticles, affect the magnitude of the surface charge density. Namely, standardization of the efficiency of TENGs should be officially established. While discussing and implementing the standards for TENGs in research are essential, the cost of power generation, as governed by material and manufacturing, is also a critical factor not captured in the figure of merit alone. The necessary analysis, derived in our manuscript, optimizes the coupled triboelectric and economic problem for the device dimensions as a function of cost. This optimization yields the minimum $ per W value for triboelectric power generation and provides a framework for comparing materials beyond the figure of merit.
2) Develop effective packaging technology for TENGs.^99^ The packaging of TENGs will be vitally important to make them commercial products, especially for applications in blue energy harvesting, because moisture or any surface contaminants can greatly affect the performance of the TENGs, and seawater can short circuit the TENGs' electrodes. Considering the harsh marine environment, the packaging has to have specific attributes to be durable: the packaging materials should be noncorrosive, resistant to heat and radiation, and, preferably, chemically inert. Furthermore, such packaging is more difficult to realize than conventional packaging because TENGs are devices that convert mechanical energy into electricity. The flexibility and elasticity of the packaging materials, as well as the packaging strategy, need to be carefully considered to protect the devices from salt water without reducing the mechanical triggering too much. State‐of‐the‐art technologies that utilize a rolling ball in a waterproof container might be a good solution. However, thousands of such TENG units need to be connected to form networks to provide large‐scale power generation, and waterproofing at the cracks and joints is also essential. Hence, current packaging techniques still have serious problems that require further research. For example, packaging in the case of water environments and harsh environments should be investigated because it may affect the performance of a TENG. Moreover, other parameters, such as humidity, can affect TENG performance.^100^
3) Find a suitable approach to power management and energy storage. Energy harvesting from the environment is subject to variations in the environment and is time‐dependent and unstable, but a power source with fixed output voltage and current is required to drive conventional electronics. Therefore, storing the generated energy in a battery or capacitor is essential so that it can be used to power a device in a regulated manner. Furthermore, the output characteristics of TENGs are a high voltage but a low current compared to those of SCs or thermoelectric cells. Hence, transformers are often used to regulate the output performance and the impedance of TENGs. However, due to the nonperiodic and low‐frequency mechanical triggering involved in blue energy harvesting, traditional transformers produce a considerable power loss in power management. Between the power generation TENG units and the storage unit, proper power management approaches are needed to boost the output current with minimum power losses. Moreover, for a network of TENGs to harvest blue energy at large scales, an interconnection strategy among TENGs is essential. Since TENGs typically have very high voltages (≈100 V) and low currents (from tens to hundreds of µA), multiple TENGs in the network should be connected in parallel such that the total current of the network is the sum of the individual currents of the TENGs. The output of TENGs is strongly dependent on the load resistance. Therefore, for maximum power transfer from a TENG network, a PMM is indispensable.

**Figure 13:**
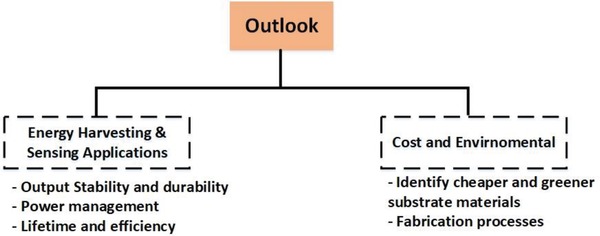
A representative outlook on the major factors that need to be addressed while designing and optimizing TENG‐based systems for energy harvesting and sensing applications.

The recommended research directions for academic researchers and industrial entrepreneurs over the coming years are as follows (**Figure** **13**):

Several TENG fabrication processes should be investigated and compared. The comparison should include the TENG output power, efficiency, fabrication process steps, complexity, cost, size, reliability, MTTF (mean time to failure), fabrication yield, and process variations.

Several materials should be explored in the TENG fabrication process to provide distinct fabrication recipes along with the material costs. This exploration is critical from an economic perspective because a developed country can afford moderately priced materials with higher efficiency, whereas developing countries might be more interested in fabricating inexpensive material–based TENGs at the expensive of some degraded efficiency.

The TENG design parameters, the AC–DC converter following the TENG, the DC–DC converter, the maximum power point tracking (MPPT) circuit, and the battery regulator should be co‐optimized to maximize the overall TENG system efficiency.

Self‐powered sensing networks of micro to macrosized TENGs represent a significant milestone within the context of healthcare, smart city infrastructure, power monitoring, security surveillance, and industrial fabrication. The features of TENGs make them a highly integrative technology with minimal fabrication costs, from rigid to flexible/stretchable TENG devices. Additional research must account for TENG operation under extreme environments and loading conditions.

Although TENGs have emerged as an efficient approach to harvesting mechanical energy, their robustness and lifetime have yet to be improved through an effective and widely applicable method. Developing protocols and techniques for investigating the long‐term stability and realizable tests for IoT applications is crucial. The main degradation mechanism of TENGs is widely accepted to be wear resulting from mechanical motion during operation. In our manuscript, we highlighted the impact of reducing wear and abrasion on TENGs. This reduction has a direct correlation with the lifetime of the TENG device, which may need to be maintained and preserved to ensure optimum working conditions.

The power density remains the most critical parameter that defines the final application of TENGs and will determine whether TENGs can be used in the power generation market. As of today, TENGs have demonstrated power densities as high as 500 W m^−2^. While this is excellent for many applications, further improvements are still required for this technology to be competitive. We believe that the interface/contact region mainly controls the power density. Thus, exploring the interfacial area with the goal of understanding some essential parameters from the molecular to macroscopic levels, such as the interfacial chemistry, interfacial zone, and contact design, is necessary. In this review article, we addressed different conventions and components for moving triboelectrification forward by controlling the interfacial properties of the materials at the nanoscale, such as by expanding the charge mobility, the dynamic interface and actuated molecular dipole moments. We hope that this discussion will provide some guidelines for students and researchers working in this field to improve the power density of TENGs.

Meanwhile, challenging and potential topics remain to be explored to establish a real blue energy farm based on TENG devices, such as plastic pollution, cable connections, the climate relationship, the environmental durability, and the ecological influence. The current research for TENG devices is in the early stage compared to other energy fields. Therefore, we should further discuss and continuously study these topics in the future.

## 12 Conclusions

TENG‐based technology could provide a wide range of devices for multiple applications. This technology is quite efficient and eco‐friendly, with the capability for harvesting energy from a wide range of sustainable energy sources. Moreover, the role of TENGs in smart self‐powered IoT applications has been the main subject of this review, where a plethora of comprehensive studies have attested to their superior performance and compatibility with other energy harvesting technologies for hybridized designs. These attributes have been discussed here for possible smart city designs and modern urban planning, which are subjects of great interest in this new era of IoT. The technology is still new, and much room for improvement exists under proposed principles for the development and standardization of existing TENG‐based technologies. This review aims to provide an innovative research direction in exploring TENGs as a promising solution for harnessing the abundant and underexplored blue energy for advanced self‐powered technologies to meet the exponentially rising energy demands. The literature has shown amazing accomplishments in the field of large‐scale energy harvesting and wireless network devices operated by TENG‐based technologies. Moreover, we studied the progress of SCPUs that can be realized by coupling TENGs with energy storage devices, such as batteries and capacitors. Then, we introduced different designs of power management circuits, supercapacitors, and batteries that can be combined with TENG devices. Finally, we covered the major factors for enhancing TENG‐based systems for energy harvesting and sensing applications.

## Conflict of Interest

The authors declare no conflict of interest.
